# Significance of genetic polymorphisms in long non-coding RNA *AC079767*.*4* in tuberculosis susceptibility and clinical phenotype in Western Chinese Han population

**DOI:** 10.1038/s41598-017-01163-y

**Published:** 2017-04-19

**Authors:** Zhenzhen Zhao, Mei Zhang, Jun Ying, Xuejiao Hu, Jingya Zhang, Yanhong Zhou, Yi Zhou, Xingbo Song, Binwu Ying

**Affiliations:** 1 Department of Laboratory Medicine, West China Hospital, Sichuan University, Chengdu, 610041 P.R. China; 2 Department of Clinical Laboratory, Santai People’s Hospital, Santai, Sichuan 621100 P.R. China

## Abstract

Recent studies have implicated long non-coding RNA, *AC079767*.*4*, as a highly susceptible gene in tuberculosis. The aim of the study was to preliminarily explore the possible association of single nucleotide polymorphisms (SNPs) in *AC079767*.*4* gene with clinical phenotypes and TB susceptibility in Western Chinese Han population. The improved multiplex ligation detection reaction (iMLDR) method was employed to genotype 4 SNPs in *AC079767*.*4* in 554 tuberculosis patients and 561 healthy individuals. In subgroup analysis, only the C allele for rs12477677 was associated with the decreased susceptibility to pulmonary TB with a *p*-value of 0.026, but *p*-value was 0.103 after Bonferroni correction. In total samples, haplotype [ACAC], representing four *AC079767*.*4* variants, was found to slightly decrease TB risk (*p* = 0.045). Furthermore, patients with the CC genotype of rs12477677 were correlated with fewer occurrences of fever (*p* = 0.016), while patients carrying the T allele were associated with lower levels of ESR in the dominant model of rs1055229 (*p* = 0.021). For the first time, we reported the potential susceptibility and clinical traits of tuberculosis with lncRNA variants in the Western Han Chinese population. Our data indicate *AC079767*.*4* polymorphisms may potentially act as novel biomarkers for tuberculosis diagnostic and therapeutic purposes.

## Introduction

Tuberculosis (TB) caused by *Mycobacterium tuberculosis* (MTB) infection presents a major threat all over the world. According to the WHO Global Tuberculosis (TB) report in 2015, 9.6 million incident cases of TB and 1.5 million TB-related deaths are estimated to occur worldwide^1^. China ranks second among the 27 high TB-burden countries issued by WHO, which accounts for nearly 11% of the global burden. It is estimated that approximately a fifth of the population is infected with MTB across China; however, only 5–10% of infected individuals will develop active TB disease during their lifetime^2^. Abundant evidence from the investigations of case observations, twin studies and mouse models suggests that host genetic factors are of particular importance for determining susceptibility to TB disease^3^. For years, many genetic loci have been identified to be associated with TB development, including genetic variants of the Wnt signaling pathway^4^, cytokines and chemokines^5^, and toll-like receptors^6^. Recently, there has been considerable work revolving around genetic determinants of disorder clinical phenotypes. Pan G. *et al*. have previously shown that known multiple sclerosis (MS) risk single nucleotide polymorphisms (SNPs) could influence conversion to MS, relapse and disability progression, suggesting MS genetic risk variants may significantly influence the clinical course of MS^7^. Therefore, better understanding of the roles of genetic polymorphisms in mechanisms underlying TB disease and how these variants affect the clinical phenotypes of TB may benefit diagnosis as well as the development of treatments and vaccines.

Systemic genomic analysis has demonstrated that most of the human genome is transcribed; however, very little of it has protein-coding capacity, thus producing a broad range of non-coding RNA transcripts (named as ncRNA)^8^. Long non-coding RNAs (lncRNAs) are the largest class of ncRNA transcripts, which are defined as transcripts more than 200 nucleotides in length^9^. Recently, there has been an increase in attention around lncRNAs and their roles in human development and disease. LncRNAs have been known to be important regulatory players of gene expression through functioning as protein scaffolds, transcription co-activators or inhibitors, and mRNA decoys or microRNA sponges at transcriptional or post-transcriptional levels^10, 11^. By dysregulating the target gene, lncRNA could participate in the development and progression of many human diseases, including cancer^12^, cardiovascular disorders^13^, neurodegenerative diseases^14^, and autoimmune diseases^15^. Previous reports have shown that lncRNAs are also essential in human infectious diseases such as TB. A study by Pawar K. *et al*. revealed that down-regulated lncRNA MEG3 caused enhanced eradication of intracellular infected mycobacteria in *Mycobacterium bovis* BCG-infected macrophages^16^. Importantly, according to microarray analyses conducted by Yi Z. *et al*., the long non-coding RNA transcript AC079767.4 (Ensembl ID ENST00000429730) encoded by gene *AC079767*.*4* (Ensembl ID ENSG00000224137) was differentially expressed in the active tuberculosis group, latent tuberculosis infection (LTBI) group and healthy control group. AC079767.4 transcript was down-regulated in both the active TB group and LTBI group compared with healthy controls, and its expression level was elevated in the LTBI group when compared to the active TB group^17^. These researchers subsequently verified these microarray data using RT-qPCR method and the results had excellent concordance with the microarray data. It is possible that the lncRNA *AC079767*.*4* gene may be involved in the establishment and progression of TB infection. Inspired by the aforementioned information, we assume that *AC079767*.*4* would be a highly promising susceptibility gene in TB.

With the rapid growth of identifications of cancer-associated lncRNAs, considerable efforts have concentrated on studying the contribution of lncRNA genetic variants in susceptibilities to many types of human cancers. Liu Y *et al*.^18^ found that the rs7763881 variant in lncRNA *HULC* was highly up-regulated in hepatocellular carcinoma and may confer susceptibility to HBV-related hepatocellular carcinoma in Chinese populations. In addition to disorder risk, several studies focused on the potential association between lncRNA polymorphisms and both disease clinical phenotype and treatment response. For example, lncRNA *CASC8* polymorphism rs10505477 has been identified to be greatly correlated with platinum-based chemotherapy response in lung cancer in the dominant model^19^. Bayram S. *et al*. found that the rs12826786 TT genotype of well-characterized lncRNA *HOTAIR* was markedly related with multiple clinic-pathological features of breast cancer, such as larger tumor size (T3 and T4), distant metastasis (M1), advanced TNM stage (III and IV), and poor histological grade (III), which are all related to worse cancer progression^20^. These finding strongly indicate that lncRNA polymorphisms can act as new molecular biomarkers in the diagnosis and therapeutic areas of human diseases. However, to date there is a lack of genetic evidence describing the effect of lncRNA polymorphisms on clinical traits and predisposition to TB in the Han Chinese population. Thus, our study genotyped a set of SNPs within *AC079677*.*4* among 554 tuberculosis cases and 561 healthy controls and systematically analyzed the relationship between risk as well as the clinical characteristics of active tuberculosis disease and selected lncRNA polymorphisms.

## Results

### General characteristics of the study subjects

The demographic and clinical features of the study participants are described in Table 1. Overall, there was no significant difference in age and gender between cases and controls, and both groups mainly involved middle-aged males. Among patients with tuberculosis, smoker and BCG scar were 53.97% and 52.17%, respectively, while smoker and BCG scar of healthy controls were 39.93% and 42.78%, respectively (*p* all <0.001). The mean values of body mass index (BMI) for the control and TB groups were 23.53 and 20.74 kg/m^2^, respectively (*p* < 0.001). For laboratory examinations, when compared to control subjects, patients with active tuberculosis showed an obvious increase in the levels of C-reactive protein (CRP), erythrocyte sedimentation rate (ESR), and absolute leucocyte, platelet and monocyte counts (*p* all <0.001). TB patients had reduced levels of albumin (Alb), hemoglobin (Hb) and erythrocyte counts (*p* all <0.001) compared to healthy controls.

**Table 1:** General characteristics of the study subjects.

Characteristics	TB^a^ (n = 554)	HC^b^ (n = 561)	*P*
**General data**			
Age, mean ± SD (years)	43.25 ± 19.87	44.23 ± 11.49	0.417
Gender (males)	343/211 (61.91%)	331/230 (59.00%)	0.351
BMI (kg/m^2^)	20.74 ± 2.53	23.53 ± 2.31	**<0.001**
BCG scar n (%)			**<0.001**
Yes	289 (52.17)	240 (42.78)	
No	200 (36.10)	250 (44.56)	
Uncertain	65 (11.73)	71 (12.66)	
Smoking n (%)			**<0.001**
Smoking	299 (53.97)	224 (39.93)	
Ever Smoking	94 (16.97)	95 (16.93)	
Nonsmoking	161 (29.06)	242 (43.14)	
**TB subtype n (%)**			
PTB^c^	275 (49.64)		
EPTB^d^	62 (11.19)		
PTB & EPTB^e^	217 (39.17)		
**Laboratory examinations**			
Albumin (g/L)	34.55 ± 6.65	46.72 ± 2.60	**<0.001**
Erythrocyte (×1012/L)	4.14 ± 0.79	4.85 ± 0.46	**<0.001**
Hemoglobin (g/L)	115.85 ± 23.47	147.26 ± 15.13	**<0.001**
Platelets (×109/L)	250.40 ± 68.10	171.12 ± 49.07	**<0.001**
Leucocytes (×109/L)	7.57 ± 3.49	6.11 ± 1.30	**<0.001**
Monocytes (×109/L)	0.62 ± 0.35	0.35 ± 0.12	**<0.001**
C-reactive protein (mg/L)	19.65 (6.04–69.13)	5.43 (1.79–18.42)	**<0.001**
ESR (mm/h)	46.00 (22.00–74.50)	20.36 (8.45–56.81)	**<0.001**
Positive TB-DNA n (%)	212 (38.26)	—	—
Positive smear n (%)	162 (29.24)	—	—
Positive culture n (%)	134 (24.18)	—	—
**Chest image of TB (n = 337) n (%)**			
Cavity lesion	70 (20.77)		
No cavity lesion	267 (79.23)		
**Systemic symptoms n (%)**			
Fever	295 (53.25)		
Loss weight	217 (39.17)		
Night sweat	176 (31.77)		
Poor appetite	232 (41.88)		
Fatigue	188 (33.94)		
**Local chest symptoms n (%)**			
cough	312 (56.32)		
hemoptysis	89 (16.06)		
Chest pain	207 (37.36)		
dyspnea	44 (7.94)		

Annotation: TB = tuberculosis; HC = healthy controls; PTB = pulmonary tuberculosis; EPTB = extra-pulmonary tuberculosis; PTB & EPTB = pulmonary tuberculosis combined with extra-pulmonary tuberculosis.

A series of TB-associated laboratory indices were also collected in the current study, mainly including tuberculosis subtype and etiological examinations. Amongst the TB cases enrolled in the study, 49.64% (275/554) were pulmonary tuberculosis (PTB), 11.19% (62/554) were extra-pulmonary tuberculosis (EPTB) and 39.17% (217/554) were PTB combined with EPTB (PTB&EPTB). Among patients having EPTB, the proportion of tuberculosis pleurisy was the largest with 25.09% (70/279), the followings were bronchial tuberculosis and tuberculous meningitis, with the ratios of 22.22% (62/279) and 14.34% (40/279), respectively. The positive rate for TB-DNA was 38.26% (212/554), 29.24% (162/554) for the smear microscopy and 24.18% (44/182) for culture, implying that TB-DNA testing was more sensitive than both smear microscopy and culture for diagnosing TB (38.26% versus. 29.24% and 24.18%, respectively). In a cohort of patients with pulmonary tubercular lesion, most of which received chest radiographic examinations read by professional physicians on admission, we found that lung cavitation lesions accounted for 20.77% (70/337) of all patients enrolled. In terms of the clinical presentations of TB patients, fever was the most common constitutional clinical symptom (53.25%) in our studied cases, followed by complaint of poor appetite (41.88%). Meanwhile, the most frequent local symptom was cough (56.32%).

### LncRNA genetic polymorphisms in relation to tuberculosis risk

*Genotyping results*. Genotyping of selected SNPs was successfully completed for all 554 TB patients and 561 healthy controls. Genotype distributions of 4 SNPs within the *AC079767*.*4* gene in the control group did not deviate from Hardy-Weinberg equilibrium (HWE) (*p* > 0.05 for all loci). Detailed information with respect to genotyped SNPs, including chromosomal locations, functional consequences, minor allele frequencies and *p*-values for HWE test in controls is summarized in Table S1.

#### Single SNP association analysis

Table 2 displays the genotype distributions and allelic frequencies of the four SNPs in the *AC079767*.*4* gene between all tuberculosis cases and controls. The minor allele (C allele) frequency of rs12477677 was 46.48% in case group and 50.53% in controls, and the *p*-value of 0.056 after adjusting for age and gender was borderline significant (OR = 0.85, 95% CI = 0.72–1.00), but the *p* value was 0.224 after adjustment with Bonferroni correction. The frequency of genotype rs12477677 CC in cases was 22.02% and 25.13% in controls. The presence of allele C in homozygosis was more common in controls, although the difference did not reach statistical significance (*p* > 0.05). On the basis of results shown in Table 2, we surprisingly found that polymorphism rs10178277 was in perfect linkage disequilibrium with rs1055228 (*r* ^*2*^ > 0.99). The other three loci (rs10178277, rs1055228 and rs1055229) showed no significant differences between cases and controls in either genotype or allele frequencies (all *p* greater than 0.05). We next carried out a genetic model analysis, including dominant and recessive models, to further explore the difference in genotype distributions. The particular data are exhibited in Table 3. No significant differences in the genetic pattern analysis were demonstrated. However, we observed that rs12477677 was associated with non-significant declined risk for TB infection in the dominant model (CC + CT *versus* TT), with an estimated OR of 0.77 (95% CI = 0.59–1.01, *p* = 0.059 after adjusting for age and sex, *p* = 0.237 after Bonferroni correction).

**Table 2:** Association between genetic polymorphisms and TB risk in *AC079767*.*4*.

SNP	Variant	Case n (%)	Control n (%)	OR (95% CI)	P*	P**	Variant	Case n (%)	Control n (%)	*P*
rs10178277	G	361 (32.58)	339 (30.21)	1.12 (0.93–1.35)	0.219		GG	55 (9.93)	45 (8.02)	0.435
A > G	A	747 (67.42)	783 (69.79)	1	—		GA	251 (45.31)	249 (44.39)	
							AA	248 (44.76)	267 (47.59)	
rs12477677	C	515 (46.48)	567 (50.53)	0.85 (0.72–1.00)	**0**.**056**	**0**.**224**	CC	122 (22.02)	141 (25.13)	0.136
T > C	T	593 (53.52)	555 (49.47)	1	—		CT	271 (48.92)	285 (50.81)	
							TT	161 (29.06)	135 (24.06)	
rs1055228	G	361 (32.58)	339 (30.21)	1.12 (0.93–1.35)	0.219		GG	55 (9.93)	45 (8.02)	0.435
A > G	A	747 (67.42)	783 (69.79)	1	—		GA	251 (45.31)	249 (44.39)	
							AA	248 (44.76)	267 (47.59)	
rs1055229	T	230 (20.76)	214 (19.07)	1.12 (0.90–1.38)	0.311		TT	22 (3.97)	16 (2.85)	0.311
C > T	C	878 (79.24)	908 (80.93)	1	—		TC	186 (33.57)	182 (32.44)	
							CC	346 (62.46)	363 (64.71)	

P*: *P* value was calculated using logistic regression analysis after adjusting for age and gender.

P**: *p* value after Bonferroni correction.

*P*: *p* value was calculated by Chi-square test.

**Table 3:** Analysis of *AC079767*.*4* genetic variants relevant to TB risk in Chinese Han population.

SNP	Dominant Model			Recessive Model		
	OR (95% CI)	P*	P**	OR (95% CI)	P*	P**
rs10178277 A > G	1.12 (0.89–1.42)	0.344		1.26 (0.84–1.91)	0.266	
rs12477677 T > C	0.77 (0.59–1.01)	**0**.**059**	**0**.**237**	0.84 (0.64–1.11)	0.221	
rs1055228 A > G	1.12 (0.89–1.42)	0.344		1.26 (0.84–1.91)	0.266	
rs1055229 C > T	1.10 (0.86–1.41)	0.435	—	1.41 (0.73–2.712)	0.305	

P*: *P* value after adjusting for gender.

P**: *p* value after Bonferroni correction.

Considering the correlation between age of onset of tuberculosis and genetic variants, described by Hijikata M. *et al*.^21^, we performed an age-stratified analysis in our study. Unfortunately, we did not see any association of all 4 SNPs with predisposition to TB in our age-subgroup analysis (data not shown).

To examine whether these four candidate SNPs were preferentially correlated with specific tubercular subtype according to Fernando S.L. *et al*.’s study^22^, we classified all tuberculosis patients into 2 subgroups: 275 patients with PTB and 217 with PTB&EPTB. Due to the low number of samples, EPTB was excluded from the subgroup analysis. The results are summarized in Table 4. Rs12477677 seemed to have a stronger magnitude of decreased risk for PTB in comparison to all forms of TB, and subjects carrying the C allele were correlated with decreased susceptibility to PTB relative to T allele carriers (*p* = 0.026, OR = 0.79, 95% CI = 0.64–0.97). But the relationship was also non-significant via Bonferroni correction with *p* = 0.103. However, the remaining three SNPs were not substantially different in the PTB subgroup analysis (data not shown). Additionally, genotypes of 4 polymorphisms showed no significant association with PTB susceptibility by applying dominant and recessive patterns. When comparing genotype distributions between PTB&EPTB and healthy controls, no any significant results were obtained (data not shown).

**Table 4:** Comparison of polymorphism distributions of *AC079767*.*4* in PTB subpopulation.

SNP	Variant	Case n (%)	Control n (%)	OR (95% CI)	P*	P**	Variant	Case n (%)	Control n (%)	*P*
rs10178277	G	190	339	1.23 (0.98–1.53)	0.071	0.285	GG	35	45	0.086
A > G	A	360	783	1	—		GA	120	249	
							AA	120	267	
rs12477677	C	246	567	0.79 (0.64–0.97)	**0**.**026**	**0**.**103**	CC	56	141	0.073
T > C	T	304	555	1	—		CT	134	285	
							TT	85	135	
rs1055228	G	190	339	1.23 (0.98–1.53)	0.071	0.285	GG	35	45	0.086
A > G	A	360	783	1	—		GA	120	249	
							AA	120	267	
rs1055229	T	111	214	1.07 (0.83–1.39)	0.591		TT	16	16	0.079
C > T	C	439	908	1	—		TC	79	182	
							CC	180	363	

P*: *P* value was calculated using logistic regression analysis after adjusting for gender.

P**: *p* value after Bonferroni correction.

*P*: *p* value was calculated by Chi-square test.

#### Haplotype analysis

**Figure 1:**
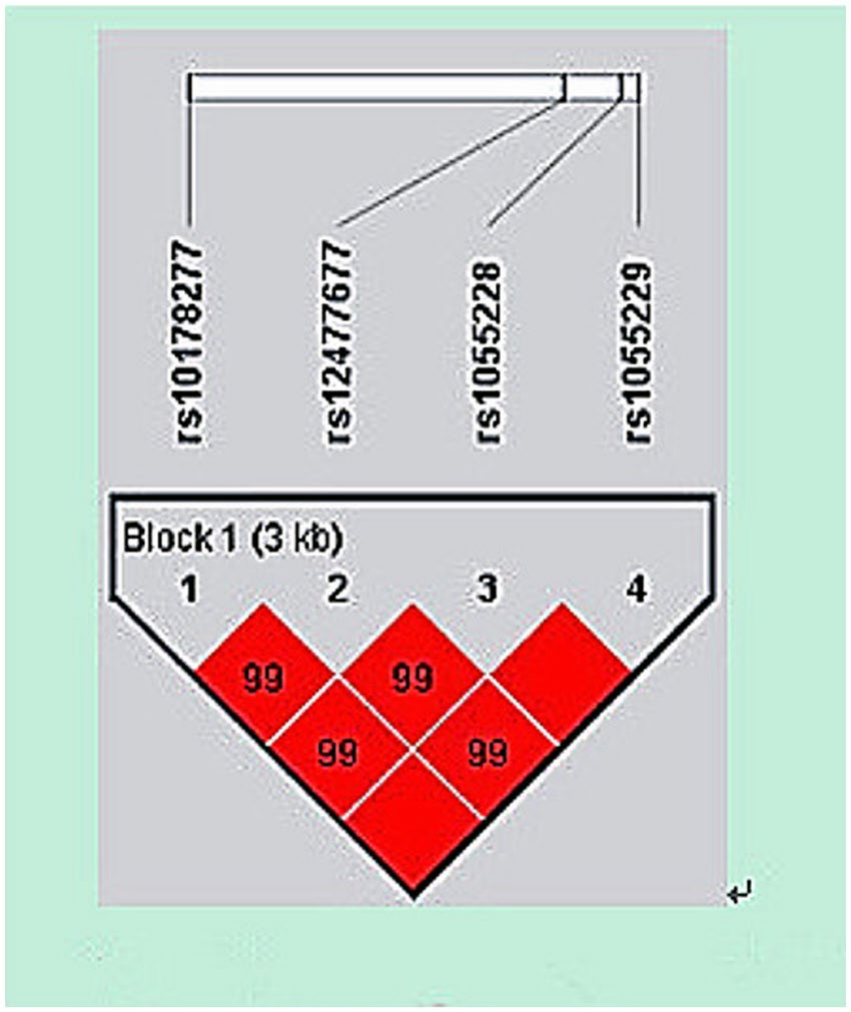
Linkage disequilibrium (LD) plot of 4 SNPs of lncRNA *AC079767*.*4*. Strong LD is represented by a high linkage disequilibrium correlation coefficient and a red square.

The linkage disequilibrium measurement and haplotype construction of variants of the *AC079767*.*4* gene were conducted in the present study. Figure 1 presents that four loci in the *AC079767*.*4* gene were in one linkage disequilibrium (LD) block based on the threshold of pairwise *r* ^*2*^ > 0.80. Three haplotypes, ACAC, GTGC, and ATAC, were constructed for *AC079767*.*4* and those consisted of rs10178277, rs12477677, rs1055228, and rs1055229. Table 5 summarizes the haplotype frequencies as well as their associations with tuberculosis predisposition. The results revealed that the haplotype ACAC was significantly associated with reduced susceptibility to TB at a *p*-value of 0.045 with an OR of 0.84 (95% CI = 0.71–0.99).

**Table 5:** Haplotype constructions of the *AC079767*.*4* variants associated with the risk of tuberculosis.

Haplotype	Frequency			OR (95%CI)	*P*
	All	TB case	Healthy control		
ACAC	0.485	0.464	0.506	**0.84 (0.71–0.99)**	**0.045**
GTGC	0.313	0.325	0.301	1.12 (0.94–1.34)	0.210
ATAT	0.199	0.207	0.191	1.11 (0.90–1.36)	0.345

*P*: *P* value was calculated using Chi-square test.

### Association between polymorphisms and clinical phenotypes

**Figure 2:**
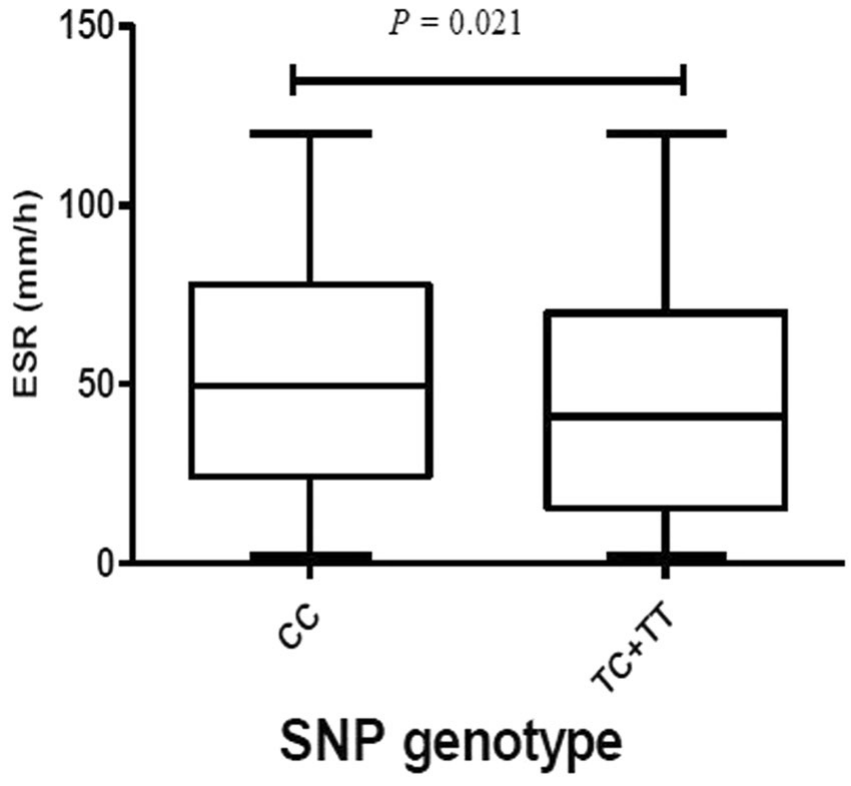
The level of ESR in relation to rs1055229 in TB patients. Rs1055229 (C > T) genotype was stratified on the basis of the dominant model, TC + TT *vs*. CC.

Early reports showed that the clinical manifestation and progression of active tuberculosis might be impacted by the given genetic polymorphism^23^. We therefore analyzed whether the four candidate SNPs influenced patients’ clinical manifestations of the disease. Common TB-associated clinical traits were evaluated, including clinical symptoms (including typically systemic and local symptoms), albumin and hemoglobin concentrations, absolute leukocyte counts, erythrocyte sedimentation rate (ESR), C-reactive protein (CRP) level and lung cavitation lesion. Regarding the possible TB susceptibility locus, rs12477677, our results revealed that patients homozygous for the minor allele (C allele) were less likely to suffer from fever, the most frequent symptom in our study patients, than genotype TT and TC carriers (*p* = 0.016, as shown in Table 6). Due to the small numbers of the homozygous minor genotypes of SNPs rs1055229 and rs10178277, these two loci were stratified using the dominant model. The results are listed in Tables 7 and 8. Although the SNP rs1055229 was not found to be significantly associated with TB susceptibility in individual SNP analysis, it was significantly associated with ESR levels routinely used to assess the disease activity for TB. As presented in Fig. 2 and Table 7, ESR levels were clearly higher among patients carrying homozygous CC genotype (median = 49.50, IQR = 24.25–77.75 mm/h) in the rs1055229 locus compared to those with TC and TT genotypes (median = 41.00, IQR = 15.50–70.00 mm/h) with a *p* value of 0.021. An increased trend for patients with the CC genotype of rs1055229 in CRP levels was found but did not reach statistical significance (*p* = 0.076). For rs10178277, none of the clinical phenotypes were correlated with the genotypes (Table 8).

**Table 6:** Association of rs12477677 polymorphism with clinical traits of patients with TB.

Characterizations	TT (N = 161)	TC (N = 271)	CC (N = 122)	*P*
**Manifestations n** (**%**)				
Systemic symptoms				
Fever	91 (57.96)	154 (58.11)	50 (43.10)	**0.016**
Weight loss	64 (40.76)	103 (38.87)	50 (43.10)	0.734
Night sweat	51 (32.48)	86 (32.45)	39 (33.62)	0.973
Poor appetite	60 (38.22)	123 (46.42)	49 (42.24)	0.253
Fatigue	60 (38.22)	89 (33.58)	39 (33.62)	0.593
Local chest symptoms				
cough	93 (57.76)	160 (59.04)	59 (48.36)	0.129
hemoptysis	23 (14.29)	47 (17.34)	19 (15.57)	0.695
chest pain	55 (34.16)	109 (40.22)	43 (35.25)	0.39
dyspnea	12 (7.45)	24 (8.86)	8 (6.56)	0.708
**Laboratory examinations**				
Albumin (g/L)	35.99 ± 6.36	35.51 ± 6.81	35.09 ± 6.71	0.573
Hemoglobin (g/L)	117.96 ± 22.68	120.14 ± 24.07	121.73 ± 23.18	0.396
Leucocytes ( × 109/L)	7.21 ± 3.53	7.21 ± 3.33	7.47 ± 3.79	0.773
C-reactive protein (mg/L)	14.05 (5.48–62.82)	18.90 (5.46–72.45)	27.10 (9.98–76.50)	0.242
ESR (mm/h)	43.50 (22.00–68.75)	45.00 (18.50–74.50)	54.50 (23.00–81.50)	0.264
**Chest image n** (**%**)				
Cavity formation	17 (17.53)	25 (15.53)	20 (28.17)	0.07

Annotation: 82 patients lacked CRP results and 49 lost ESR data.

**Table 7:** Association of rs1055229 polymorphism with clinical traits of patients with PTB using the dominant model.

Characterizations	CC (N = 346)	TC + TT (N = 208)	*P*
**Manifestation n** (**%**)			
Systemic symptoms			
Fever	176 (52.54)	119 (58.62)	0.199
Weight loss	142 (42.39)	75 (36.95)	0.247
Night sweat	115 (34.33)	61 (30.05)	0.352
Poor appetite	147 (43.88)	85 (41.87)	0.714
Fatigue	117 (34.93)	71 (34.98)	>0.99
Local chest symptoms			
cough	189 (54.62)	123 (59.13)	0.3
hemoptysis	49 (14.16)	40 (19.23)	0.116
chest pain	137 (39.60)	70 (33.65)	0.19
dyspnea	29 (8.38)	15 (7.21)	0.741
**Laboratory examinations**			
Albumin (g/L)	35.27 ± 6.68	36.04 ± 6.58	0.869
Hemoglobin (g/L)	119.32 ± 22.58	120.73 ± 24.90	0.218
Leucocytes ( × 109/L)	7.46 ± 3.79	6.96 ± 2.91	0.105
C-reactive protein (mg/L)	20.90 (7.59–78.60)	17.10 (4.77–57.10)	0.076
ESR (mm/h)	49.50 (24.25–77.75)	41.00 (15.50–70.00)	**0.021**
**Chest image n** (**%**)			
Cavity lesion	50 (23.47)	20 (17.24)	0.238

Annotation: 82 patients lacked CRP results and 49 lost ESR data.

**Table 8:** Association of rs10178277 polymorphism with clinical traits of patients with PTB using the dominant model.

Characterizations	AA (N = 248)	AG + GG (N = 306)	*P*
**Manifestation n** (**%**)			
Systemic symptoms			
Fever	125 (52.74)	170 (56.48)	0.437
Weight loss	98 (41.35)	119 (39.53)	0.736
Night sweat	76 (32.07)	100 (33.22)	0.849
Poor appetite	102 (43.04)	130 (43.19)	>0.99
Fatigue	84 (35.44)	104 (34.55)	0.901
Local chest symptoms			
cough	132 (53.23)	180 (58.82)	0.187
hemoptysis	36 (14.52)	53 (17.32)	0.437
chest pain	91 (36.69)	116 (37.91)	0.837
dyspnea	16 (6.45)	28 (9.15)	0.312
**Laboratory examinations**			
Albumin (g/L)	35.51 ± 6.82	35.60 ± 6.52	0.472
Hemoglobin (g/L)	122.51 ± 23.94	177.69 ± 22.90	0.817
Leucocytes ( × 109/L)	7.17 ± 3.34	7.35 ± 3.61	0.402
C-reactive protein (mg/L)	24.20 (6.90–70.20)	17.40 (5.48–65.20)	0.459
ESR (mm/h)	48.00 (19.00–78.00)	44.00 (24.00–72.00)	0.82
**Chest image n** (**%**)			
Cavity lesion	31 (20.39)	39 (22.03)	0.82

Annotation: 82 patients lacked CRP results and 49 lost ESR data.

## Discussion

Our study aimed to preliminarily investigate the possible associations of susceptibility and disease traits in active tuberculosis with four candidate SNPs in lncRNA *AC079767*.*4* within the Western Chinese Han population. The main findings of our study are that not only target lncRNA polymorphisms (rs12477677) are possibly related with risk for TB development but also that SNPs (rs12477677 and rs1055229) may influence clinical TB disease presentations. This study strongly suggests that genetic variants in *AC079767*.*4* could become promising novel biomarkers for diagnosis and therapeutic applications in TB.

Currently, more non-coding genetic loci and protein-coding genes associated with TB have been determined. Non-protein coding RNA transcripts are diverse and, with a cutoff value of 200 nucleotides, can be roughly classified into small non-coding RNAs and long non-coding RNAs (lncRNAs). The idea that non-coding RNAs can be regulatory molecules of gene expression has been well established for many years, especially for small non-coding RNAs like microRNAs (miRNAs). In contrast to miRNAs, the roles of lncRNAs in diverse human physiological and pathological processes remain largely elusive. Recently, compelling studies have already shown that many lncRNAs are implicated in the host response against *Mycobacterium tuberculosis* (MTB) infection. For example, a study by Yang W. *et al*. uncovered that lncRNA-CD244, up-regulated by CD244 signaling, could inhibit IFN-γ/TNF-α expression by mediating repressive chromatin states at *infg*/*tnfa* loci through epigenetic programming in CD8^+^ cells infected with active tuberculosis^24^. The critical roles of lncRNA genetic variants in development and prognosis of various human cancers have been adequately studied. However, there is a limited amount of data supporting the effects of lncRNA genetic polymorphisms on predisposition and clinical phenotype for TB, which has an especially high prevalence in China. Given this concern, we performed the case-control study in the Western Chinese Han population to preliminarily explore potential TB-associated SNPs within lncRNA *AC079767*.*4* and to assess whether these loci influence clinical manifestations of tuberculosis.

According to single locus analysis, there were no significant associations of TB risk with all 4 SNPs within *AC079767*.*4* between tuberculosis cases and control subjects in the Western Chinese Han population. Nonetheless, the differences in both C allele frequencies (*p* = 0.056) and genotype distributions under the dominant model (*p* = 0.059) in the rs12477677 locus neared statistical significance. In subsequent subgroup analysis, we discovered the C allele for rs12477677 was statistically associated with reduced susceptibility to pulmonary tuberculosis (PTB) (*p* = 0.026) when comparing patients with only PTB to healthy individuals. However, statistical results showed that the allele and genotypic distributions were similar when cases with PTB & EPTB were compared to PTB patients and control individuals, respectively. Although the differences found in the rs12477677 locus did not remain statistical significant after Bonferroni correction, based on our results, we speculate that the C allele of rs12477677 within the lncRNA *AC079767*.*4* gene seems to possibly pose a relatively weak protective effect for the development of tuberculosis. However, this genetic component by itself may not be a determining factor for severe clinical types of TB, such as PTB and EPTB combined. Subsequently, we conducted haplotype analysis for these genetic variants and revealed that the ACAC haplotype, formed by 4 *AC079767*.*4* polymorphisms and the possible beneficial C allele in the rs12477677 locus, was statistically associated with decreased susceptibility to TB disease (*p* = 0.045). This finding was consistent with the individual SNP rs12477677 analysis. Our data indicate the potentially protective effect of the C allele in rs12477677 in TB development.

Although statistical analyses of the single locus of other 3 *AC079767*.*4* variants failed to yield significant correlations, the investigations of these polymorphisms supplemented our knowledge on the association of lncRNA polymorphisms with TB susceptibility. In our study, the detected SNPs were mainly concentrated on intrinsic regions. Thus, polymorphisms in exons and regulatory genetic sequences might have been ablated, which may further the need for more comprehensive and systematic variants association studies in the future. Consequently, the correlation between this lncRNA gene and TB should be interpreted cautiously.

**Figure 3:**
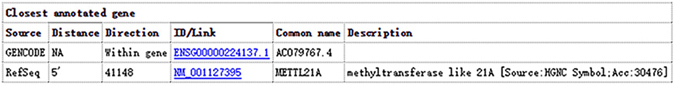
The genomic location of SNP rs12477677. The HaploReg v4.1 (http://www.broadinstitute.org/) determined the location.

The lncRNA *AC079767*.*4*, aberrantly expressed among the LTBI, active TB and healthy control groups, is from an intergenic region adjacent to the protein-coding gene *methyltransferase like 21 A* (*METTL21A*). The polymorphic locus rs12477677 was found to lie in the *AC079767*.*4* intron region and reside in the *METTL21A* downstream regulatory interval by the bioinformatics software HaploReg v4.1 (http://www.broadinstitute.org/), as shown in Fig. 3. It was reported that lncRNAs and nearby protein-coding genes might share local transcriptional and up-stream regulation effects^25, 26^. A possible explanation is that rs12477677 might serve as a protective SNP through influencing the expression or function of the *AC079676*.*4* and *METTL21A* genes. Although there is currently a lack of report regarding the biologic functions of lncRNA *AC079767*.*4*, the protein product METTL21A of the *METTL21A* gene is well-described and defined as a novel human protein methyltransferase (MTase) responsible for trimethylation of the conserved lysine residue found in several Hsp70 (heat shock protein of ~70 kDa)^27^. Additionally, it has been established that several members of the Hsp70 family are closely associated with tuberculosis^28^. Together, our findings provide useful information for the future investigation of potential biological functions of SNP rs12477677 in tuberculosis development.

Using the cohorts of tuberculosis patients with clinical data, we investigated whether the four candidate SNPs in *AC079767*.*4* were associated with clinical presentations of active TB. We found that rs12477677 and rs1055229 loci significantly contributed to differences in the clinical manifestations of active tuberculosis. Our analysis showed that patients carrying the homozygous CC genotype of rs12477677 had a highly significant association with fewer frequency of fever (*p* = 0.016) among patients with TB, which to a certain extent reflects the host inflammatory response to pathogen invasion. This is consistent with the result from the genetic susceptibility association analysis, further supporting the possibly favorable impact of the C allele in rs12477677 on TB risk as well as clinical presentation form. One interesting observation in our study was that the effect of SNP rs1055229 on TB clinical features and its insignificant effect on TB susceptibility differed. The TB non-susceptibility locus rs1055229 was linked to ESR levels in patients with TB within the current dataset. The elevated ESR levels were strongly associated with the CC genotype carriers when compared to those with the T allele, including the homozygous and heterozygous genotypes (*p* = 0.021). Hence, we consider that the variants in lncRNA *AC079767*.*4* are most likely related to inter-individual differences in host defense response to MTB infection. Previous studies have shown that rs11465802 and rs1884444 in *IL-23R* were significantly associated with the drug-resistance of the infected MTB strain and cavitary lesion among pulmonary tuberculosis patients, respectively, and were unassociated with susceptibility to pulmonary TB in Chinese Uygurs^29^. The differences between TB susceptibility SNPs and genetic loci related to TB clinical phenotype indicate that TB development, progression and disease manifestation may be independently impact by different genetic loci and different signaling pathways. Although the detailed molecular mechanisms are poorly understood, these results may support the argument that lncRNA genetic variants may influence host defense responses against MTB infection and lead to different clinical manifestations in patients with the active disease.

This investigation represents the first exploration of the association between lncRNA genetic variations and active TB in a Han Western Chinese population. In our disease association study, we recruited all participants from the same geographic area during the same study period to minimize the bias of MTB pathogen exposure. However, our study has several limitations. First, we did not further carry out the gene-environment or gene-gene interaction analyses in our study because of the uncorrelated-TB risk among these SNPs. Second, we only studied a small amount of SNPs within lncRNA *AC079767*.*4*. Third, the relatively small sample size restricts the possibility of determining a significant genetic association. Lastly, we did not distinguish the specific population with latent tuberculosis infection (LTBI) among the disease-free controls, and thus the correlation of lncRNA variations with LTBI remains uncharacterized. Therefore, replication studies in other large independent populations and ethnicities, along with functional experiment studies, are urgently needed to explain the mechanisms of genetic polymorphisms in lncRNA *AC079767*.*4* in TB development and clinical manifestation.

In conclusion, the potentially TB-associated protective effect was first identified for the C allele of SNP rs12477677 in lncRNA *AC079767*.*4* in our study. Furthermore, we observed that the rs12477677 CC genotype may protect patients with TB from bearing fever, while the rs1055229 CC genotype was associated with greater ESR levels in a dominant model. Our findings strongly indicate that lncRNA *AC079767*.*4* polymorphisms may serve as novel molecular biomarkers to diagnose tuberculosis and even as potential therapeutic targets.

## Methods

### Study subjects

**Figure 4:**
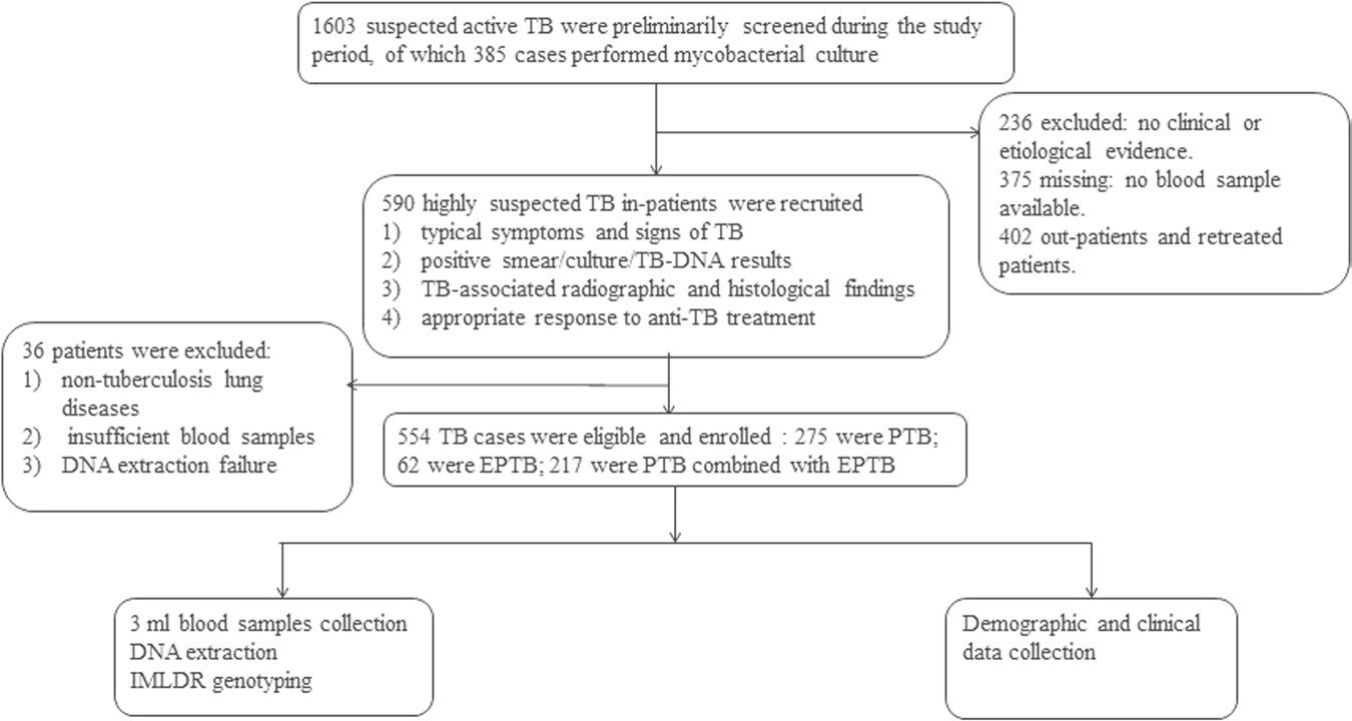
The flow diagram of our study. Demographic information and clinical data was reviewed from the medical system of West China Hospital of Sichuan University.

This case-control association study included 554 tuberculosis patients and 561 healthy controls. All subjects were recruited from West China Hospital of Sichuan University between November 2011 and September 2015. All enrolled patients were newly diagnosed with TB, confirmed by two independent experienced respiratory physicians. The active TB diagnosis was made based on the following criteria: typical symptoms and signs of active TB disease, positive smear/culture/TB-DNA results, TB-associated radiological and histopathological findings as well as appropriate response to anti-TB treatment. Patients with HIV-infection, immunodeficiency disease, hepatitis virus infection, and other lung diseases were excluded from this study. The flow diagram of enrollment of TB patients was delineated in Fig. 4. Healthy controls were recruited from a pool of a health physical examination population. To be a healthy individual, people needed to be asymptomatic with no history of TB and negative sputum smear and culture. All controls have normal chest radiograph. Control groups were frequency-matched with case groups regarding age and gender, and all participants were of unrelated Han ethnicity.

Demographic data and clinical information of the studied population were reviewed from the medical record system of West China Hospital of Sichuan University. Three milliliter (ml) EDTA-anticoagulated whole blood samples were collected from each subject for genotyping. This study was approved by the Clinical Trials and Biomedical Ethics Committee of West China Hospital, Sichuan University (reference no. 198; 2014) and written informed consent was obtained from all the participants. All methods in this study were carried out in accordance with the approved guidelines.

### Single nucleotide polymorphisms selection and genotyping

The human *AC079767*.*4* gene is located on chromosome 2q34 and is approximately 4.6 kb nucleotides long with two exons. The genetic polymorphism data of the entire sequence of *AC079767*.*4* was obtained from the dbSNP database (http://www.ncbi.nlm.nih.gov/projects/SNP/). First, all SNPs were initially filtered according to the minor allele frequency (MAF) of 0.05 or more in Han Chinese in Beijing (CHB). Subsequently, we preferentially selected the SNPs that were located in potentially functional regions, including promoter, exon, untranslated region (UTR), intron and potential regulatory regions. Combined with experimental conditions required for genotyping, four SNPs in *AC079767*.*4* (rs10178277, rs12477677, rs1055228 and rs1055229) were eventually included in our analysis.

Genomic DNA was isolated from peripheral blood samples by using QIAamp® DNA blood mini kit (Qiagen, Germany) according to the manufacturer’s instructions. The improved multiplex ligation detection reaction (iMLDR) method (Genesky Biotechnologies Inc., Shanghai, China) was used to genotype candidate SNPs, as described elsewhere^30^. The primer information for the multiplex PCR reaction is described in Table S2. The oligos and probes used in the ligation reaction are presented in Table S3. Briefly, this genotyping assay consisted of an initial multiplex PCR reaction that was designed to amplify fragments containing all 4 SNP loci, followed by the highly specific improved ligation reaction. Finally, a 0.5 *μ*l ligation product was fractionated and loaded in ABI 3730XL, and the GeneMapper v4.1 software was employed to analyze the raw data. The different SNPs were identified according to different extended lengths at the 3′ terminal site, and the specific allele of each SNP was distinguished by different fluorescent labels of allele-specific oligo probe pairs.

To guarantee the genotyping quality, the ddH_2_O was considered to be the negative control used in each reaction, and the genotyping experiment was blinded so that the experimentt staff was unaware of the status of case-control. Furthermore, approximately 10% of the total samples were selected at random to genotype in duplicate and the concordance rate was perfect (100%).

### Statistical analysis

The needed sample size was determined using PASS Statistical Software version 11 before data collection^31^ using the following parameters: a = 0.05, 80% power, a polymorphism prevalence of 5%, an allelic odds ratio for TB of 2 compared with the control, and a match ratio of 1:1. The Chi-square test and the Mann-Whitney U test were applied for categorical variables and continuous variables analyses, respectively. Hardy-Weinberg equilibrium (HWE) for all SNPs in the controls was assessed using the goodness-of-fit Chi-square test. Statistical methods above were performed by SPSS version 19.0 (IBM, Chicago, USA). Associations between candidate SNPs and TB risk were determined based on the distributions of allele and genotypic frequencies along with two different genetic models (dominant and recessive models). The strength of association was estimated with odds ratios (ORs) and 95% confidence intervals (CIs) using unconditional logistic regression analysis adjusted for age and gender by PLINK v1.07^32^. The Bonferroni method was further employed to correct for multiple testing. Additionally, we employed Haploview software version 4.2 to establish the linkage disequilibrium block with a threshold of the pairwise *r* ^2^ coefficient ≥0.80 and to construct haplotypes by using the expectation-maximization clustering algorithm. The correlations of each genetic variant with clinical features of tuberculosis were evaluated. All statistical tests were two-sided, and *p* < 0.05 was considered statistically significant.

## Electronic supplementary material

Supplemetary information
