# Supplementary material for: Significance of genetic polymorphisms in long non-coding RNA AC079767.4 in tuberculosis susceptibility and clinical phenotype in Western Chinese Han population
Source: Sci Rep. 2017 Apr 19;7:965. doi: 10.1038/s41598-017-01163-y (PMC5430418; doi:10.1038/s41598-017-01163-y)
Supplement: Supplementary file 1 — Supplemetary information [file 41598_2017_1163_MOESM1_ESM.pdf]

Significance of genetic polymorphisms in long non-coding RNA *AC079767.4* in tuberculosis susceptibility and clinical phenotype in Western Chinese Han population

Zhenzhen Zhao [1,*], Mei Zhang [1,*], Jun Ying[2,*], Xuejiao Hu [1], Jingya Zhang [1], Yanhong Zhou [1], Yi Zhou [1], Xingbo Song [1], Binwu Ying[1]

Table S1. Characteristics of the studied 4 SNPs.

SNP	Chr: position	Functional	MAF	HWE-*P*
rs10178277	2:208528314	Intron variant	0.314	0.231
rs12477677	2:208531122	Intron variant	0.485	0.736
rs1055228	2:208531553	Exon variant	0.314	0.231
rs1055229	2:208531683	Exon variant	0.199	0.274

Annotation: Chr: chromosome;

HWE-*P*: *P* value of Hardy-Weinberg equilibrium tests in the control group.

Table S2. Primer sequences of target SNPs for the multiplex PCR reaction.

SNPs	Primer	SequenCes (5'→3')
rs10178277	F	ACCCCAACCTGGGCAACA
	R	CCAAGGGAGTGGTGGAGTGAGA
rs12477677	F	CCAGGCAGAGAGACACTCACTGC
	R	TTTCTCATCTAGATCAAGGGGCTACAA
rs1055228	F	CCAGTGGCTTCTCGTTGCAGAT
	R	TGAGAACACGCCTCCAAATCAA
rs1055229	F	CCAGTGGCTTCTCGTTGCAGAT
	R	TGAGAACACGCCTCCAAATCAA

Table S3. The probe sequence in probe mixture.

Probe Name	Target Allele	Probe Sequence (5' phosphorated)
rs10178277FA	A	pTGTTCGTGGGCCGGATTAGTTGGGCAACAGAGCAAAACTGCA
rs10178277FG	G	pTCTCTCGGGTCAATTCGTCCTTTGGGCAACAGAGCAAAACTACG
rs10178277FP		TCTCAAATTAATTAATTAATTAATTAATAAGAATAAGATTTTTTTTTTT
rs12477677FC	C	pTTCCGCGTTCGGACTGATATCAAAGTAAGGACTCAACGAGTTCAGCTGTC
rs12477677FT	T	pTACGGTTATTCGGGCTCCTGTCAAAGTAAGGACTCAACGAGTTCAGCTGTT
rs12477677FP		ATTATTTTTTAAAAAGTATTTTCTGTATTGTAGCCCTTTTTTTT
rs1055228RA	A	pTACGGTTATTCGGGCTCCTGTACACAGCAGTAGTCAGAAAGAACACTTTCCTT
rs1055228RG	G	pTTCCGCGTTCGGACTGATATACACAGCAGTAGTCAGAAAGAACACTTTCTTC
rs1055228RP		TGTTGAATCCATTTTTCCTCTATCTCTTCTTTTTTT
rs1055229RC	C	pTCTCTCGGGTCAATTCGTCCTTGAGAACACGCCTCCAAATCAAACTTG
rs1055229RT	T	pTGTTCGTGGGCCGGATTAGTGAGAACACGCCTCCAAATCAAACCTA
rs1055229RP		TGTGTTGTCTTAATGATTAAAATAGTCTCTTGTTTTTTTTTT
